# The genetic landscape of congenital neutropenia in Poland: summary of the nationwide screening campaign

**DOI:** 10.3389/fimmu.2025.1688208

**Published:** 2025-11-26

**Authors:** Katarzyna Bąbol-Pokora, Weronika Dobrewa, Marta Bielska, Szymon Janczar, Joanna Madzio, Aleksandra Jaworowska, Sylwia Kołtan, Marcin Hennig, Joanna Renke, Iwona Dachowska-Kałwak, Magdalena Cienkusz, Wojciech Młynarski

**Affiliations:** 1 Department of Pediatrics, Oncology and Hematology, Medical University of Lodz, Lodz, Poland; 2 Department of Pediatrics, Hematology and Oncology, Collegium Medicum in Bydgoszcz, Bydgoszcz, Poland; 3 Department of Pediatrics, Hematology and Oncology, Medical University of Gdansk, Gdansk, Poland; 4 Clinical Department of Pediatric Bone Marrow Transplantation, Oncology and Hematology, Wroclaw Medical University, Wroclaw, Poland; 5 Department of Hematology, Oncology and Transplantology, Children’s University Hospital in Lublin, Lublin, Poland

**Keywords:** ELANE, neutropenia, NGS, SBDs, immunodeficiencies, CYN, SCN

## Abstract

This work summarizes 15 years of genetic research on neutropenia in the Polish pediatric cohort, explores the distribution and spectrum of disease-causing genetic variants associated with congenital neutropenia in Poland, and demonstrates the impact of a nationwide information campaign on increasing the efficiency of patient recruitment. The study included 126 patients with suspected congenital neutropenia recruited in 2008–2019 and 291 patients recruited in 2020–2023 as part of the FixNet project, which featured a nationwide information campaign. Molecular analyses were performed using Sanger sequencing (91 patients) and targeted next-generation sequencing (NGS) (326 patients) in a panel of neutropenia-related genes. The information campaign significantly increased the number of referred patients from 10.5 per year to 72.75 per year. Based on the results obtained, 102 patients belonging to 80 different families were diagnosed with severe congenital neutropenia (SCN) and neutropenia-related syndromes, the majority (43%) of whom harbored variants in the *ELANE* gene, including 12 novel ones. Most of the remaining cases were *SBDS*, *CLPB*, *SRP54*, and *CXCR4* gene defects. This work describes the largest cohort of genetic variations associated with suspected congenital neutropenia (CN) in Poland and is an important contribution to the international SCN registry.

## Introduction

Severe congenital neutropenia (SCN) is a rare immunodeficiency disorder characterized by impaired granulopoiesis. Patients are at risk of severe life-threatening infections and clonal progression to myeloid neoplasms. The prevalence of congenital neutropenia (CN) is estimated to be one to two cases per 1,000,000 live births (1). CN is caused by germline pathogenic variants in genes related to the growth, development, and differentiation of granulocytes, leading to abnormalities in neutrophil maturation, survival, and function and neutropenia in peripheral blood (2).

Since the late 1980s, significant advances have been made in the understanding of the molecular basis of CN and patient treatment. Genetic testing is crucial and actionable in patients in whom primary neutropenia is suspected. A negative family history does not exclude a genetic basis for neutropenia, and many hematopoiesis-related genetic variants have variable penetrance, leading to different disease phenotypes within a family (3). So far, at least 11 types of severe congenital neutropenia, as well as numerous syndromes associated with chronic neutropenia, have been recognized (4). The Department of Pediatric Hematology and Oncology of the Medical University of Lodz has been involved in the genetic diagnosis of neutropenia since 2008. During the first 8 years, we used Sanger sequencing as the diagnostic tool for detecting variations in *ELANE* and other CN-related genes. In subsequent years, corresponding with technological progress, we implemented commercially available and subsequently custom-designed next-generation sequencing (NGS) panels, which enabled the sequencing of more than 50 CN and chronic neutropenia-related genes. In November 2019, we initiated a nationwide congenital neutropenia awareness campaign associated with a research endeavor titled “Fix Neutropenia (FixNet): focusing on neutrophil proteases defects which serve as novel diagnostic and therapeutic options”. Our promotional efforts for the project targeted pediatric hematology and immunology centers, clinicians, patients, and parents through various channels, including radio broadcasts, medical websites, and scientific conferences.

The following results compare the effectiveness of recruitment before and after the campaign and summarize 15 years of genetic screening for neutropenia in Poland.

## Methods

### Subjects

The study included 417 enrolled patients with a physician-driven suspicion of CN. Inclusion in the study required meeting at least two of the listed criteria and was at a discretion of the referring physician: 1) persistent absolute neutrophil count (ANC) below 1,500/µL, 2) duration of neutropenia >6 months, 3) recurrent infections (>2 per year), and 4) occurrence of symptoms characteristic of neutropenia-associated syndromes (e.g., albinism, short stature, external pancreatic insufficiency, hearing loss, or metabolic abnormalities).

The study was carried out with written informed consent from all participants in accordance with the recommendations of the Medical University of Lodz Ethics Committee (RNN/353/19/KE). The anonymity of the patients was guaranteed by codifying data entry. The following data describing the clinical course of neutropenia were collected: age at diagnosis (years), gender, ANC, granulocyte-colony stimulating factor (G-CSF) treatment, bone marrow examination to check maturation arrest, hematopoietic stem cell transplantation (HSCT), transformation to myelodysplastic syndrome (MDS)/acute myeloid leukemia (AML), and outcome. The effectiveness of patient recruitment was assessed based on demographic data for the pediatric population (aged 0–19 years) from 16 Polish voivodeships covering the years 2008–2023, obtained from the depository of the Central Statistical Office, http://stat.gov.pl.

### Diagnostic methods

#### Sanger sequencing

PCR followed by Sanger sequencing was used as a molecular analysis method between 2008 and 2015. Nine CN-related genes were sequenced, i.e., *ELANE*, *GFI1*, *HAX1*, *JAGN1*, *CSF3R*, *G6PC3*, *GATA2*, *CXCR4*, and *SBDS*, in accordance with the congenital neutropenia panel at that period.

Genomic DNA was extracted from peripheral blood samples of patients using a Genomic Maxi AX Kit (A&A Biotechnology, Gdansk, Poland) and checked for quality using Qubit v.3 (Thermo Fisher Scientific, Waltham, Massachusetts, USA).

Whole genes were screened using specific primers (**Supplementary Table S1**) designed by means of the Primer3 tool v. 0.4.0. The PCR and clean up were carried out following standard protocols. Sanger sequencing was performed on an ABI 3130 four-capillary sequencer (Thermo Fisher Scientific, USA), and the results were analyzed using Sequencher v. 5.0.

#### Next-generation sequencing

Targeted NGS was performed using either the TruSight One panel (Illumina Inc., San Diego, CA, USA) (2016–2017) or the custom-designed SureSelect QXT panel (Agilent Technologies Inc., Santa Clara, CA, USA), which included over 700 genes related to hematological and immune-related diseases. Sequencing libraries were prepared according to the manufacturer’s protocols (5, 6). The samples were checked for quality using Qubit v.3 (Thermo Fisher Scientific Inc.). High-throughput sequencing was performed on NextSeq 550 (Illumina Inc.) in the process of 300-bp paired-end run using the Mid Output Kit (Illumina Inc.). The data analyses of the target regions were performed using the Burrows-Wheeler Aligner Genome Alignment software and the GATK Variant Caller algorithms and mapped to the human genome reference sequence GRCh37/hg19 (7). The results were next analyzed using Variant Studio v. 3.0 (Illumina Inc.) and Integrative Genomics Viewer v.2.3 (8), and panel analysis focused on the 54 genes related to SCN and neutropenia-associated syndromes (**Supplementary Table S2**). The filtering criteria included coverage with at least 20 reads and a minor allele frequency (MAF) below 0.01 in the GnomAD database. All filtered variants were investigated using several bioinformatics tools: SIFT, Mutation Taster, and PolyPhen-2 (9–11). The pathogenicity of the revealed variants was estimated based on the ClinVar, ExAC, OMIM, HGMD, and Varsome databases (12–16) according to American College of Medical Genetics and Genomics (ACMG) classification rules (17). An internal database was also used to filter out the recurrent variants.

Patients with pathogenic or likely pathogenic variants, along with their family members, additionally underwent Sanger sequencing. Patients with no variant identified (NVI) underwent further analysis in an expanded panel of genes related to inborn errors of immunity and hematological disorders (**Supplementary Table S3**). Patients carrying a single variant in a gene related to the recessive mode of neutropenia or presenting severe bone marrow failure phenotypes underwent single-nucleotide polymorphism (SNP) array analysis.

#### SNP array

Copy number variation (CNV) identification was performed using the CytoScan HD assay (Thermo Fisher Scientific), which contains 1.9 million non-polymorphic probes and 700,000 SNP probes. The analysis was performed using the GeneChip Scanner 3000 according to the current CytoScan HD protocol. Final analysis was performed using the Chromosome Analysis Software (ChAS) v. 4.3 with a 10-kbp CNV detection size filter.

### Statistical analysis

The Statistica Software v. 13.3 (StatSoft, Cracow, Poland) was utilized for all analyses. Categorical variables are presented as numbers and percentages and were compared using the χ^2^ test or Fisher’s exact test. Continuous variables are presented as medians followed by interquartile ranges (IQRs) and were compared using the Mann–Whitney U test to analyze differences between two groups. A p-value below 0.05 was considered statistically significant.

## Results

### Impact of FixNet campaign

**Figure 1:**
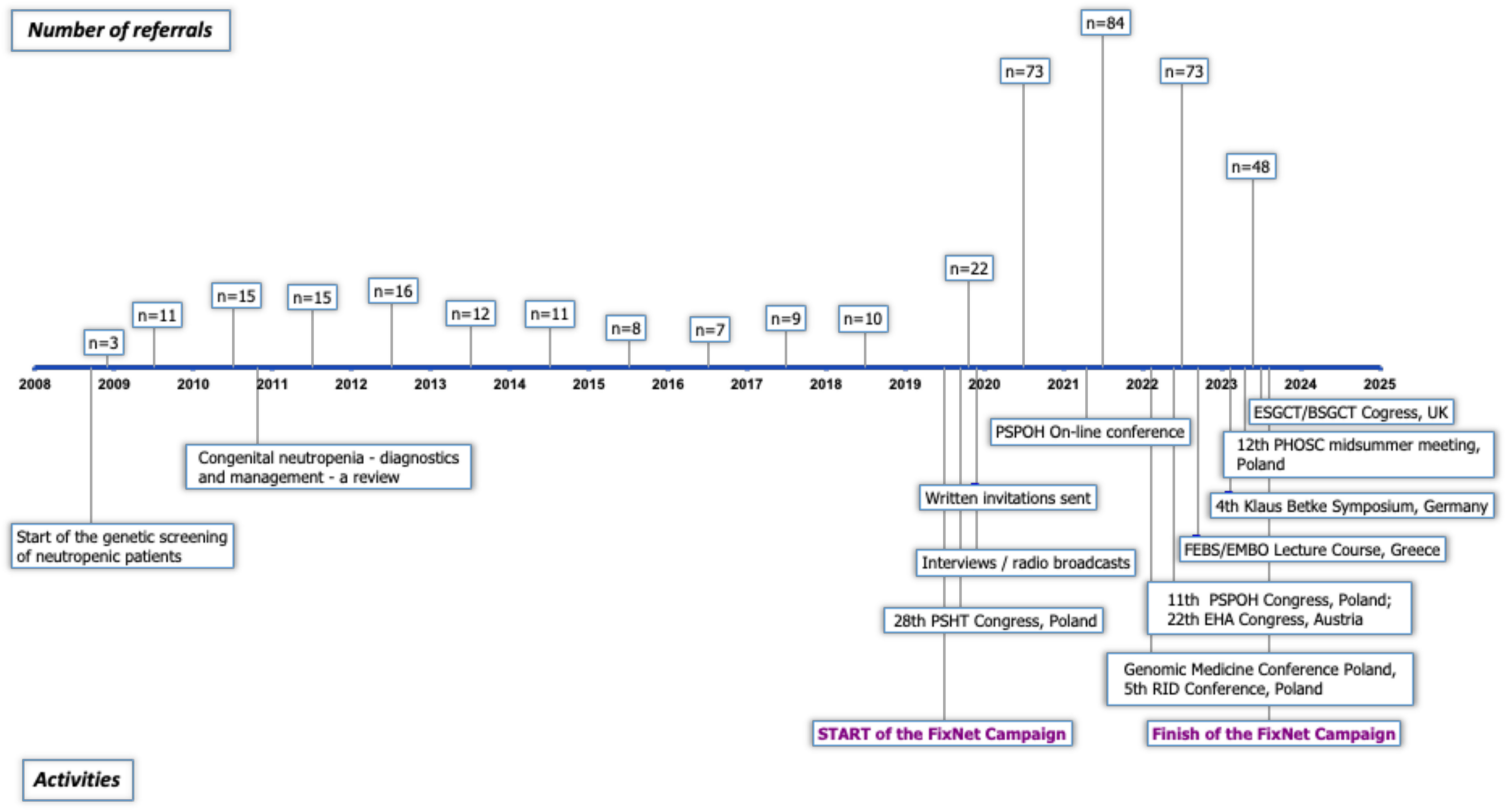
Timeline of promotional activities carried out during the FixNet project. Timeline includes the number of referrals per year since the commencement of genetic screening. PSHT, Polish Society of Haematologists and Transfusiologists; PSPOH, Polish Society of Paediatric Oncology and Haematology; RID, rare immune diseases; FEBS, Federation of European Biochemical Societies; EMBO, European Molecular Biology Organization; PHOSC, Pediatric Hematology, Oncology and Stem Cell; ESGCT/BSGCT, European/British Society of Gene and Cell Therapy.

In 2008, our recruitment efforts began, targeting patients suspected of having CN for genetic screening. In October 2019, we launched the nationwide FixNet campaign, which began with intensive promotional activities during the 28th Congress of the Polish Society of Hematologists and Transfusiologists, which was attended by nearly 1,300 participants. We continued the campaign during the COVID-19 pandemic through online meetings and conferences, as well as through interviews in nationwide radio broadcasts. Additionally, we sent written invitations to participate in the FixNet project to hospital directors and heads of hematology, oncology, and immunology departments across Poland. We continued the campaign in 2022 and 2023 at numerous national and international conferences, where we also presented preliminary genetic results. The list of our promotional activities is presented in **Figure 1**.

**Figure 2:**
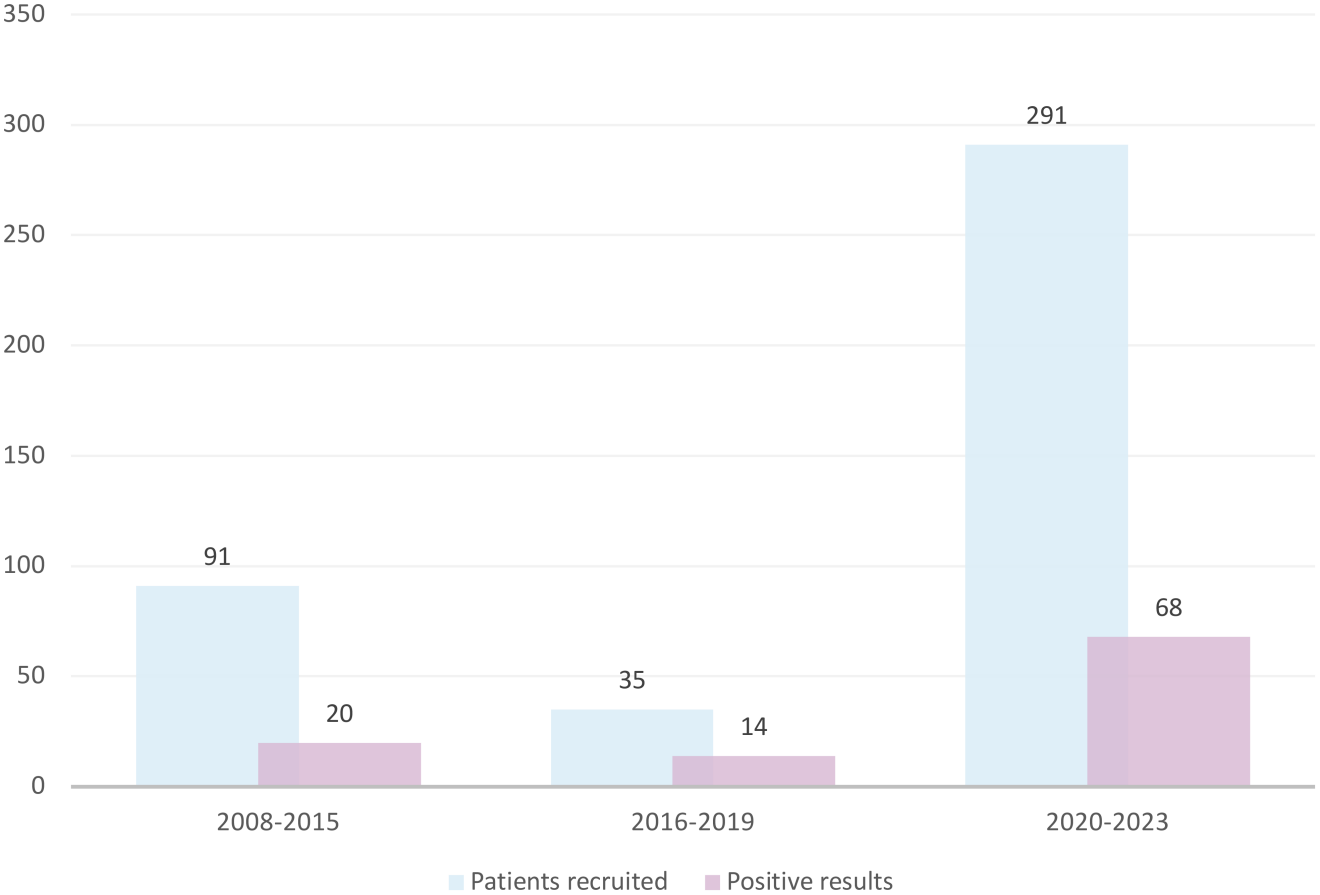
The increase in the recruitment and genetic screening efficacy of CN patients observed after introduction of the nationwide information campaign. CN, congenital neutropenia.

**Figure 3:**
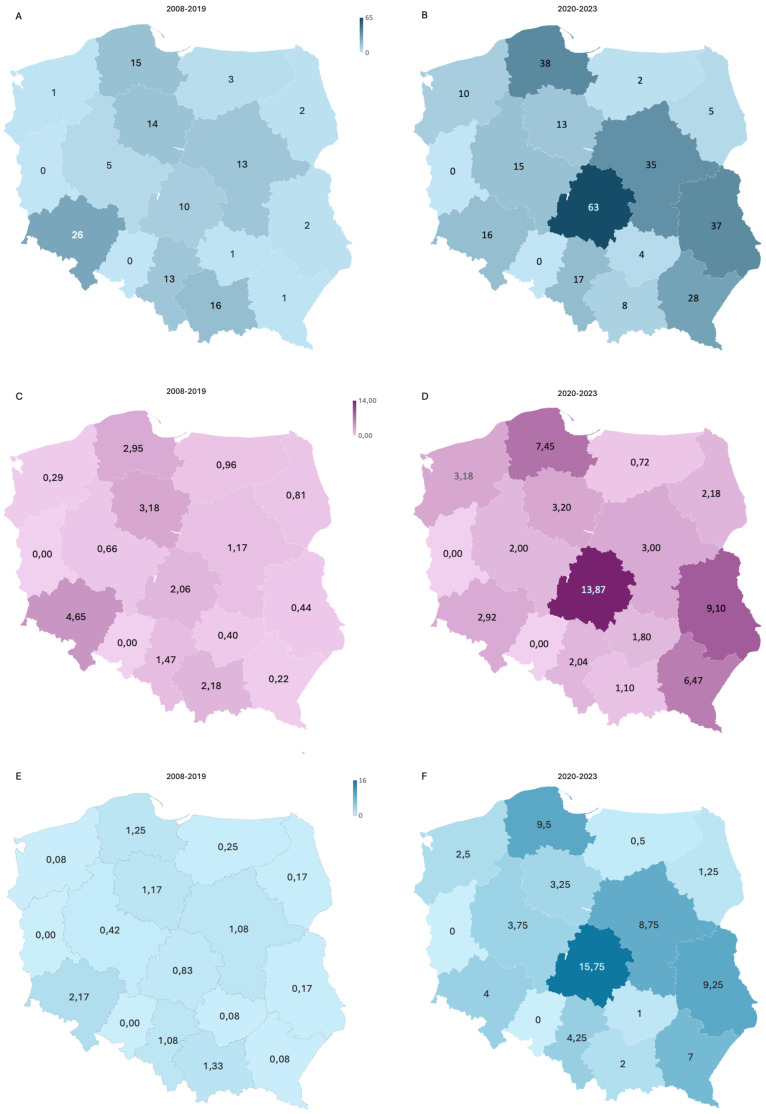
An increase in the recruitment efficiency of CN patients shown by region. A comparison of the total number of referrals **(A, B)** and the number of referrals per 100,000 children **(C, D)** during first period of CN screening (2008–2019) and after start of the FixNet project, followed by a nationwide campaign (2020–2023). **(E, F)** The number of patients recruited per year in both periods. CN, congenital neutropenia.

We had recruited 91 patients by 2015, averaging approximately 11.4 patients per year. The implementation of NGS technology in 2016 significantly expanded our diagnostic testing capacity for neutropenia; however, likely due to a lack of funding for genetic screening (available mainly on a commercial basis at that time), recruitment efficacy dropped to 8.75 patients per year. The FixNet project, which provided free genetic testing within a research project, caused an increase in the number of recruited neutropenic patients from 10.5 to 72.75 per year, reaching 291 patients in less than 4 years (**Figure 2**). We established cooperation for CN patient recruitment with 14 hematological centers in Poland and reported an increase in the number of referrals in all of them. We recorded the largest increase in the number of referrals in the Lodz province (from 2.6 to 13.87 per 100,000). We also observed the campaign’s impact on a significant increase in referrals in the Lublin (from 0.44 to 9.1 per 100,000), Pomerania (from 2.95 to 7.45 per 100,000), and Subcarpathia provinces (from 0.22 to 6.47 per 100,000) (**Figure 3**). In addition to the increase in recruitment, the success rate of genetic testing also remained satisfactory at 23.4% for causes of congenital neutropenia and 36% when including genetic findings leading to other hematological disorders. In the earlier stages of the study, the positive results were 21.9% using the Sanger method and 40% after introducing a targeted NGS panel for congenital neutropenia (**Figure 2**).

### The characteristics of the entire cohort

The study cohort included patients divided into two subgroups: patients with genetic variants identified (GVI) in neutropenia-related genes (n = 102) and those with no variants identified (NVI) in neutropenia-related genes (n = 315), as summarized in **Table 1**. The median age at referral was significantly higher in the GVI group (32 months, IQR: 6–108) compared to the NVI group (13 months, IQR: 7–36; p < 0.0090). Sex distribution was similar between the groups (p = 0.2564). Median white blood cell (WBC) and ANC values were also comparable across subgroups (p = 0.529 and p = 0.8960, respectively). However, the GVI group showed a markedly higher prevalence of family history of neutropenia (34.3% *vs*. 8.3%, p < 0.0001) and severe infections (29.4% *vs*. 8.8%, p < 0.0001) compared to the NVI group.

**Table 1:** Clinical characteristics stratified by genetic status in the study cohort.

Clinical characteristics	Entire cohort N = 417	GVI group N = 102	NVI group N = 315	p-level
Age at referral [months] Median (IQR)	16 (7–54)	32 (6–108)	13 (7–36)	0.0090*
Sex male/female N (%)	193/224 (47/53)	52/50 (51/49)	141/174 (45/55)	0.2564**
WBC [cells/µL] Median (IQR)	4,700 (3,065–6,845)	4,955 (2,673–8,043)	4,600 (3,070–6,540)	0.5290*
ANC [cells/µL] Median (IQR)	300 (130–650)	225 (83–483)	312 (135–660)	0.8960*
Family history of neutropenia N (%)	61 (14.6)	35 (34.3)	26 (8.3)	<0.0001**
Severe infections N (%)	58 (13.9)	30 (29.4)	28 (8.8)	<0.0001**
Hematological malignancies N (%)	5 (1.2)	4 (3.9)	1 (0.3)	0.0410**
Other comorbidities N (%)	55 (13.2)	14 (13.7)	41 (13)	0.7367**
HSCT N (%)	16 (3.8)	14 (13.7)	2 (0.6)	<0.0001**
G-CSF treatment N (%)	85 (20.4)	48 (47)	37 (11.7)	<0.0001**

GVI, genetic variants identified in genes associated with neutropenia; NVI, no variants identified in genes associated with neutropenia; WBC, white blood cell; ANC, absolute neutrophil count; HSCT, hematopoietic stem cell transplantation; G-CSF, granulocyte-colony stimulating factor; IQR, interquartile range.

*Mann–Whitney U-test.

**Fisher’s exact probability test.

Hematological malignancies were more common in the GVI group (3.9% *vs*. 0.3%, p = 0.0410), while rates of other comorbidities were similar between the groups (p = 0.7367). Notably, HSCT and G-CSF treatment were significantly more frequent in patients with identified genetic variants (13.7% *vs*. 0.6%, p < 0.0001; 47% *vs*. 11.7%, p < 0.0001, respectively).

### Molecular characteristics

**Figure 4:**
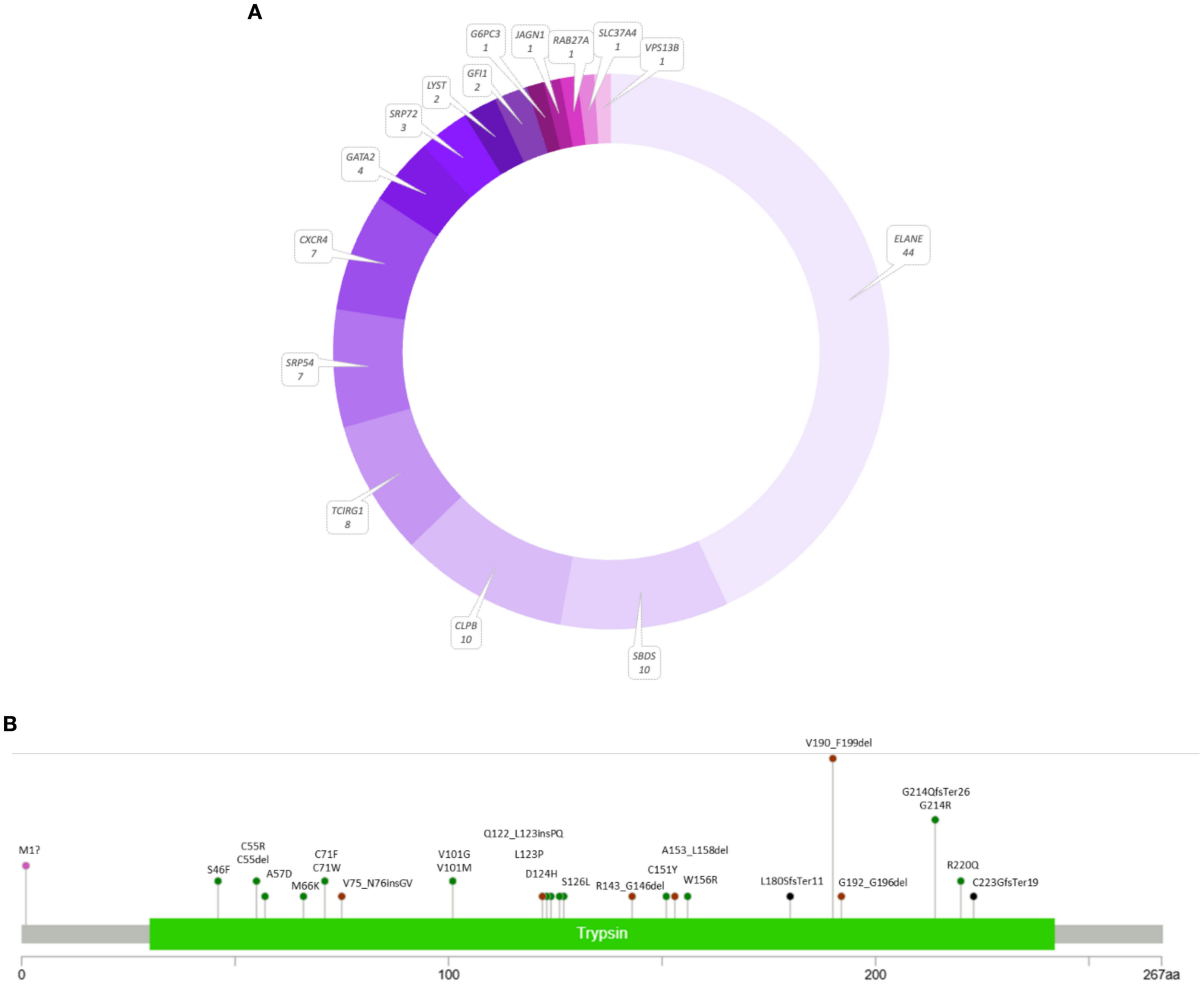
The results of the genetic screening in the panel of 54 neutropenia-related genes **(A)** and variant distribution in the *ELANE* gene **(B)** using a lollipop plot (18).

In total, 102 patients (23.4%) were positive for a germline defect leading to neutropenia. Among them, 65 patients were diagnosed with SCN or cyclic neutropenia (CyN), distributed as follows: SCN1/CyN caused by defects in the *ELANE* gene, n = 44; SCN2 (*GFI1* gene defects), n = 2; SCN4 (*G6PC3* gene defects), n = 1; SCN6 (*JAGN1* gene defects), n = 1; SCN8/Shwachman–Diamond-like syndrome (*SRP54* gene defects), n = 7; and SCN9 (*CLPB* gene defects), n = 10. Additionally, 37 patients were diagnosed with other syndromes involving neutropenia (**Figure 4A**).

Out of 44 patients from 38 families with the *ELANE* gene defect, there were 35 with a final diagnosis of SCN1 and nine with CyN. Notably, 22 patients exhibited missense variants, including the recurrent p.Gly214Arg change. Twelve patients had splice variants, including 10 affecting exon 4 splicing, resulting in an in-frame p.Val190_Phe199del. Other findings included seven patients with indels and three with start-loss changes.

Six out of nine CyN patients in our cohort had the p.Val190_Phe199del splice variant. The remaining three patients had novel in-frame deletions p.Gly192_Gly196del and p.Cys55del and a missense p.Ser46Phe, which was reported before to cause SCN1. Twelve variants have not been reported before. All splice site variants revealed in this study were known pathogenic changes confirmed by functional studies. A detailed distribution of mutations identified in our cohort within the *ELANE* gene is presented in **Figure 4B**.

The next two largest groups included patients with defects in the *SBDS* and *CLPB* genes, each consisting of 10 patients. All Shwachman–Diamond syndrome (SDS) patients belonging to eight families were compound heterozygotes, and each of them was a carrier of a splice donor variant c.258 + 2T>C, in combination with a second change: recurrent nonsense variant p.Lys62* in five patients or the novel variants p.Thr7Profs*6, p.Val36Alafs*23, p.Glu99Aspfs*21, and p.Cys119Tyr in the remaining patients. Ten patients were carriers of heterozygous *CLPB* variants, including known p.Arg297Trp and p.Arg598Cys changes, associated with isolated dominant neutropenia and a novel p.Arg599Cys variant located as well in the ATPase domain. Seven patients had heterozygous alterations in *SRP54*, five of which were recurrent in-frame p.Thr117 deletion, and two were novel variations: a deletion of exons 14 to 16 and a missense variant p.Asn503Thr. The next seven patients, including five members of one family, were diagnosed with Warts, Hypogammaglobulinemia, Infections, and Myelokathexis (WHIM) syndrome. The family shared a heterozygous frameshift variant p.Ser338Phefs*6 in the *CXCR4* gene, and three family members demonstrated symptoms of cyclic neutropenia, which constituted the initial diagnosis. The remaining two unrelated patients had nonsense variants p.Arg334* and p.Ser338*. Heterozygous variants of the *TCIRG1* gene were found in eight patients from six families, including the previously described variant p.Ala417Thr and four novel ones: p.Arg802Gln, p.Ile721Asn, p.Asn503Ser, and the splice site variant c.2118 + 5G>A.

We also identified pathogenic changes in other genes associated with hematological phenotypes, including *GATA2* (n = 4), *SRP72* (n = 3), *LYST* (n = 2), *GFI1* (n = 2), and single *SLC37A4*, *VPS13B*, *G6PC3*, *JAGN1*, and *RAB27* variants.

Overall, we found 35 novel variations in the neutropenia-related genes, which accounted for over 30% of all reported variants. Detailed results, including a list of revealed variants, references, and scores for variants of unknown significance, are presented in **Supplementary Table S4**. Cases in which no mutations were initially identified underwent reanalysis in an expanded panel of genes related to inborn errors of immunity and hematological disorders, leading to the identification of a further 42 significant variants, associated with common variable immune deficiency (CVID), immune dysregulation, or bone marrow failures, most frequently involving the *SOS1*, *TNFRSF13B*, *STAT5B*, *FAS*, and *CTLA4* genes (**Supplementary Figure S1**).

### Hematological findings in genetically positive subgroup

**Figure 5:**
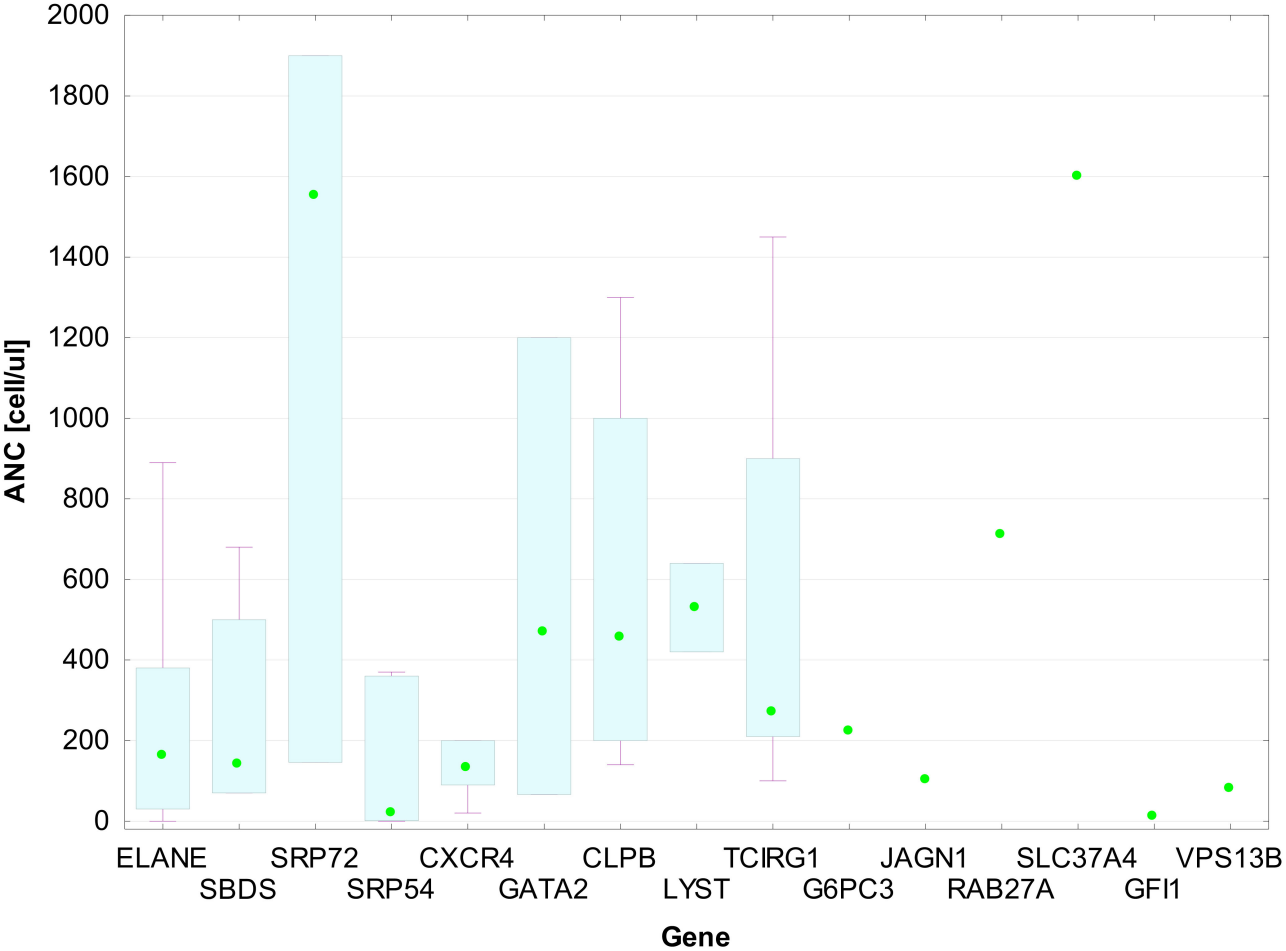
ANC medians with IQR for patient groups by gene mutation. Each box in the plot represents the interquartile range (IQR) of absolute neutrophil count (ANC) values for patients with mutations in a given gene. The bottom and top edges of the boxes correspond to the 25th (Q1) and 75th (Q3) percentiles, respectively. The horizontal line within each box shows the median ANC. The whiskers extend to the minimum and maximum values within 1.5 × IQR from the quartiles. Individual data points are shown as green dots.

The overall presenting ANC in the GVI group was 225 cells/µL (83–483). Data were missing for nine patients from the entire cohort, mainly from the first years of our research. The median presenting ANC count varied depending on the mutated gene (**Figure 5**). It was impossible to determine the median for some gene changes due to group size limitations. The lowest ANC median was observed in the group of patients with variations in *SRP54* [20 (1–360) cell/µL], *CXCR4* [130 (90–200) cell/µL], *SBDS* [140 (70–500) cell/µL], and *ELANE* [161 (30–380) cell/µL] genes. The highest ANC median was observed in *SRP72* patients [1,550 (146–1,900) cell/µL].

In addition, we compared the clinical features of two subgroups of patients with identified genetic alterations, divided into those diagnosed before (2008–2019) and during the FixNet project (2020–2023), and observed differences in clinical characteristics and treatment approaches (**Table 2**). The median age at referral was higher during the FixNet project (60 months, IQR: 8–120) compared to the earlier group (24 months, IQR: 4.5–66), although this difference was not statistically significant (p = 0.370). Similarly, the ANC was slightly higher in the FixNet group (285 cells/µL, IQR: 90.3–702.5) compared to the before-FixNet group (205 cells/µL, IQR: 92.5–387.5), but the difference did not reach statistical significance (p = 0.160). The proportion of patients with severe infections remained comparable across the two time periods (32.3% *vs*. 28%, p = 0.652). However, a significant reduction in the use of G-CSF treatment was observed in the FixNet group (33.8%) compared to the earlier group (73.5%, p = 0.0003).

**Table 2:** Comparison of clinical characteristics in the genetically positive group before and during the FixNet project.

Clinical characteristics	2008–2019	2020–2023	p-Level
Age at referral [months] Median (IQR)	24 (4.5–66)	60 (8–120)	0.3700*
ANC [cells/µL] Median (IQR)	205 (92.5–387.5)	285 (90.3–702.5)	0.1600*
Severe infections [N (%)]	11 (32.3)	19 (28)	0.6518**
G-CSF treatment [N (%)]	25 (73.5)	23 (33.8)	0.0003**

ANC, absolute neutrophil count; G-CSF, granulocyte-colony stimulating factor; IQR, interquartile range.

*Mann–Whitney U-test.

**Fisher’s exact probability test.

### Immunological characteristics

Of all patients enrolled in the study, 28% had antigranulocyte antibody testing performed, of which 55 were positive. Of these, seven patients also had genetically determined neutropenia: five of them had defects of the *ELANE* gene, and the next two had changes in *SRP72* (Pro257Arg) and *TCIRG* (Ala417Thr) genes. The anti-HNA-1 antibodies were also found in three patients harboring defects in the *FAS*, *CTLA4*, and *TBK1* genes. This part of our study has been described previously (19).

### G-CSF treatment in genetically positive subgroup

A total of 48 patients (47%) were treated with G-CSF, while 54 (53%) patients did not require treatment. Among treated patients, nine (18.8%) had a poor response to G-CSF, of whom eight had *ELANE* defects (18% of the patients with *ELANE* changes), and one had a *CXCR4* defect (14% of the patients with *CXCR4* changes). In addition, two of the treated patients (both with *ELANE* variants) took G-CSF only interventionally during infections. Patients with variants in the *CLPB* and *TCIRG1* genes did not receive G-CSF treatment.

### Malignancy in genetically positive subgroup

Three patients (3%) developed MDS or AML. Patient P73 with *CLPB*, p.Arg599Cys change, developed MDS at the age of 11, underwent HSCT, and survived, but suffered from post-transplant immune thrombocytopenia. Patient P28 (*ELANE*, p.Gly214Arg) developed AML with transformation features at the age of 3, underwent HSCT, and survived without complications. Patient P100 (*GFI1*, p.Arg310Gly) has developed AML and is now awaiting HSCT. Interestingly, her father, P101, with the same change, had Acute Lymphoblastic Leukemia (ALL) in childhood. Patients diagnosed with SCN1 and Shwachman–Diamond syndrome were regularly screened for clonal changes that increase the risk of neoplastic transformation using NGS.

### Stem cell transplantation in genetically positive subgroup

Altogether, 13 patients (13%) underwent HSCT at a median age of 74 (14–162) months. Seven of them had *ELANE* changes, two *SBDS*, and one each with variants in *LYST*, *GATA2*, *CLPB*, and *RAB27A* (**Table 3**). In three cases (described above), the indication for transplantation was the development of AML or MDS. In the remaining cases, the HSCT took place due to the serious clinical condition of the patients and resistance to G-CSF therapy.

**Table 3:** Details of patients who received HSCT.

Patient	Gene	Protein change	Age during HSCT [months]	Reason	Research period^§^
P10	*ELANE*	p.Gly214Arg	15	No G-CSF response	D
P13	*ELANE*	p.Met1?	10	Poor clinical condition^#^	D
P21	*ELANE*	p.Gly214Arg	1st 12 months 2nd 14 months	No G-CSF response	B
P24	*ELANE*	p.Gly214Arg	NA	No G-CSF response	B
P25	*ELANE*	p.Cys223Glyfs*19	NA	No G-CSF response	B
P28	*ELANE*	p.Gly214Arg	40	AML	B
P29	*ELANE*	p.Val101Met	107	Poor clinical condition^#^	B
P48	*SBDS*	p.Val36Alafs*23 p.Cys84fs*3	196	Poor clinical condition^#^	D
P51	*SBDS*	p.Lys62* p.Cys84fs*3	NA	Poor clinical condition^#^	B
P70	*GATA2*	p.Thr354Met	196	Poor clinical condition^#^	D
P73	*CLPB*	p.Arg599Cys	133	MDS	D
P84	*LYST*	p.Tyr2026* c.5214 + 3_5214 + 6del	NA	Poor clinical condition^#^	D
P98	*RAB27A*	p.Ala87Pro p.Arg184*	162	Poor clinical condition^#^	D

G-CSF, granulocyte-colony stimulating factor; AML, acute myeloid leukemia; MDS, myelodysplastic syndrome; NA, not available; HSCT, hematopoietic stem cell transplantation.

^§^The research period: B, before FixNet project started; D, during FixNet project.

^#^Poor clinical condition refers to frequent severe recurrent infections that were difficult to treat.

Patient P21 underwent two HSCTs at the ages of 12 and 14 months. The first transplant was from an unrelated donor, while the second one was a haploidentical transplantation from the mother, complicated with chronic post-transplant immune thrombocytopenia. Patient P12 is awaiting HSCT.

### Mortality in genetically positive subgroup

Overall, four patients (4%) died: P2 (*ELANE*: p.Val190_Phe199del) at 42 months, P51 (*SBDS*: p.Lys62*; p.Cys84fs*3) at 133 months, and P60 (*SRP54*: 14q13.2del, deletion of ex.14–16) at 41 months of age. The age of death of patient P30 is unknown. The cause of death of patient P2 was sepsis, while in patient P51, it was post-transplant complications. The cause of death of the remaining two is unknown.

## Discussion

This work summarizes 15 years of genetic research on neutropenia in the Polish pediatric cohort and shows the impact of a nationwide information campaign on the effectiveness of patient recruitment, resulting in a sevenfold increase. In 2008, we started recruiting patients with neutropenia for genetic tests by Sanger sequencing in the range of *ELANE*, *GFI1*, *SBDS*, *CXCR4*, *CSF3R*, *GATA2*, *HAX1*, *JAGN1*, and *WAS* genes. By 2015, we recruited 91 patients, which amounted to over 11 patients per year. In 2016, we introduced NGS technology in a range of panels consisting of over 20 genes related to neutropenia, which caused a significant increase in the outcome of positive results from 21.9% to 40%, but the recruitment efficiency dropped. To improve this, we launched the FixNet project with a nationwide advertising campaign, which led to a significant increase in the number of patients recruited, reaching almost 73 patients per year with a positive result rate of 36%. Defects associated with severe congenital neutropenia and neutropenia-related syndromes accounted for 23.4%. The cohort presented here is the largest described so far in Poland and may increase the resources of the Severe Chronic Neutropenia International Registry. A significant number of patients (n = 143) received a molecular diagnosis. Among them, there were 102 patients diagnosed with severe congenital neutropenia, cyclic neutropenia, or other neutropenia-involving syndromes. Patients with an identified genetic variant were more likely to have a positive family history (35% *vs*. 8%) and severe infections (33% *vs*. 9%), compared to the NVI patients, which highlights the clinical importance of molecular testing in affected families (3). The median age at referral was significantly higher in the GVI group (32 months) compared to the NVI group (13 months). While this may be surprising, we assume that a longer duration of symptoms and drop-out of potential immune neutropenia of infancy cases in the second year of life may explain that observation. While these points for a need for further physician education on SCN, we believe that the reduced cost of genetic diagnostics justify liberal inclusion criteria. Consistently, our group demonstrated that many patients with SCN have positive results for antibodies against neutrophils, and such patients could be lost with more stringent algorithms (19).

So far, there have been 11 types of SCN *sensu stricto* identified and numerous syndromes associated with neutropenia (4). The most common causes are defects in the *ELANE* gene, which encodes neutrophil elastase (NE) and manifests itself as an SCN1 or CyN (20). Here, we report 44 patients from 38 families with variations in the *ELANE* gene, including 12 novel variants, among whom there were 35 with a final diagnosis of SCN1 and nine with CyN. The most common change revealed is the splice variant concerning the exon 4, resulting in an in-frame deletion p.Val190_Phe199del found in nine patients belonging to six families. Among the four patients, we found p.Gly214Arg, which is known to be associated with severe phenotype resistance to GCS-F treatment and with a high risk of MDS/AML evolution (21). All p.Gly214Arg variant carriers underwent HSCT, mostly with a good outcome. Other patients that were transplanted also carried missense *ELANE* variants with a high risk for evolution to AML according to the literature, e.g., p.Val101Met (22). Missense variants, especially those disrupting bisulfide bonds and C-terminal truncation changes, are usually associated with severe phenotypes as a result of the accumulation of unfolded elastase in the Endoplasmic Reticulum (ER), in contrast to N-terminal nonsense or frameshift variants, which are known to cause complete loss of mutant *ELANE* via nonsense-mediated decay (23, 24). Variants affecting splice sites can lead to the CyN phenotype, although it is not entirely dependent on the type of variation, as was once believed (25). No frameshift or nonsense variant has been reported in CyN patients, and none of them showed the MDS/AML evolution tendency, which is in accordance with previous findings (26).

The next two largest groups included patients with defects in the *SBDS* and *CLPB* genes. All SDS patients belonging to eight families were compound heterozygotes, each carrying a common splice donor variant resulting in a premature STOP codon (27) in combination with a second pathogenic variant. Heterozygous *CLPB* alterations were located not only in the ATP domain, e.g., p.Arg598Cys, which has been described to affect mitochondrial function and induce apoptosis leading to neutropenia (28, 29), but also in the ankyrin and D2 domains, which turned out to affect CLPB oligomerization (data not shown). All patients with heterozygous *CLPB* changes had neutropenia in childhood, but ANC ​​normalized with age. The next group included seven families with heterozygous changes in *SRP54*, five of which were recurrent in-frame p.Thr117 deletion and seemed to be associated with agranulocytosis. A similar phenotype was demonstrated in a patient with a heterozygous deletion of exons 14 to 16, whereas the patient with the missense variant p.Asn503Thr showed a milder phenotype. All were ultimately diagnosed with Shwachman–Diamond-like syndrome. The heterogenic phenotypes of SDS-like disease and mild to severe neutropenia observed in patients with *SRP54* variants may be the result of the effect that mutant SRP54 proteins exert on downstream *XBP1* splicing in a dominant-negative manner (30). Seven patients, including five members of one family, were finally diagnosed with WHIM syndrome. According to the HGMD database, there are only six distinct substitution variants, five indels, and one large deletion associated with WHIM syndrome. All are truncating alterations located in the intracellular domain, which in turn cause gain of receptor function with subsequent myelokathexis (31). The family shared a heterozygous frameshift variant p.Ser338Phefs*6 in the *CXCR4* gene, and three family members had symptoms of cyclic neutropenia, which was the initial diagnosis. We also report eight patients from six families with heterozygous variants in the *TCIRG1* gene. *TCIRG1* encodes a subunit of a vacuolar H^+^-ATPase, which acts as a proton pump, and the variations in this gene are associated with autosomal recessive osteopetrosis and dominant severe congenital neutropenia (32). Some heterozygous variants were reported to reduce ANC, including the p.Ala417Thr variant found in our cohort (33). The remaining changes have unknown significance and require additional studies; however, it should be emphasized that severe neutropenia occurred in all patients and agranulocytosis in six of them. It is noteworthy that patients with a confirmed genetic variant were referred for diagnostic testing significantly later (median 24 *vs*. 13 months), which likely reflects greater phenotypic heterogeneity and limited availability of molecular testing. Similar observations were published by Dale et al., indicating that diagnostic delays in neutropenia are common and postpone the initiation of therapy (21).

Patients with no mutations identified underwent reanalysis in an extended gene panel of Inborn Errors of Immunity, which led in 42 cases to the diagnosis of neutrophil dysfunction, innate immune disorders, or autoimmunity-related syndromes such as APS1, CTLA4 deficiency, CVID, or ALPS, which may develop a more expressed phenotype with age. The CVID is thought to be the trigger of an autoimmune neutropenia, as shown in reports describing TACI variants (34, 35). CVID resulting from other gene defects can also affect the ANC and can be associated with other cytopenias with a very severe outcome (36). In addition to autoimmunity, the revealed changes also indicated bone marrow failure syndromes (dyskeratosis congenita, osteopetrosis, Diamond–Blackfan anemia, and thrombocytopenia), which are known to be a significant cause of inherited cytopenia in children (37).Our study suggests that the prevalence of congenital neutropenia in Poland is higher than it had been thought before, especially when considering the period of the FixNet campaign and the number of patients recruited. According to several national registries, the prevalence of congenital neutropenia is estimated to be 3–17 per million individuals, with *ELANE* variations as the most frequent cause of its genetic background (38–41). There is, however, an exception in the French SCN Registry, numbering 986 patients, where the most frequently mutated gene is SBDS, with a 20% representation (156 individuals). *ELANE* variation accounts for 17% of all reported defects (146 patients), *GATA2* accounts for 9%, *SLC37A4* accounts for 7%, and *TAZ*, *CXCR4*, and *VPS13B* genes account for 4%–5% of the reported variants (40). In the European section of the Severe Chronic Neutropenia International Registry (SCNIR) with 579 CN patients, *ELANE* variants contributed 26.8% (155 individuals)contributed and *SBDS* defects contributed 20.7% (120 individuals) (42). In our cohort, defects of the *ELANE* gene constituted 43% (44 individuals), and SBDS cases constituted 10% (10 individuals). *CLPB*-related neutropenia also accounted for 10% of cases, followed by defects in the *TCIRG1* (eight cases), *CXCR4* (seven cases), and *SRP72* genes (seven cases). There are also numerous reports on the genetic basis of neutropenia in various populations, demonstrating that some genetic variants are closely linked to populations of specific ancestry. In the German, Turkish, Swedish, and Iranian cohorts, a high representation of *HAX1* gene defects was found (12.3% in the European SCNIR cohort), which we did not find at all in our study. However, in the Israeli cohort of 34 CN patients, *G6PC3* variations are overrepresented and account for 18% (42, 43). *ELANE* defects have been reported so far in approximately 500 patients worldwide, mainly in two large series of 188 and 307 patients (25, 26). However, the incidence of genetically confirmed SCN/CyN reported in most countries is significantly lower.

Our study shows that a promotional campaign can significantly increase physician awareness of the occurrence of neutropenia, which ultimately has a beneficial effect on the patient, ensuring rapid diagnosis. Access to cost-free genetic testing facilitates decision-making and does not force the clinician to choose between a more symptomatic and a less symptomatic patient, which is not always reflected in the genotype. A relative scarcity of presenting and follow-up data regarding infection history, oral and organ complications, and wide laboratory studies is a major limitation of our study. This results from the fact that many referrals were motivated by the need to perform genetic studies rather than for statistical and research purposes. The patients were recruited over a long time and with varied modes of patient referral and data collection. The study presents our diagnostic experience over a long period of economic, social, and technological changes, especially pronounced in Poland. We still believe that this is a valid experience that gradually helps us develop more comprehensive approaches. We are currently setting up a national Inherited Bone Marrow Failure Registry funded by the Polish Agency for Medical Research. We would not reach that point without the technological and clinical experience we are presenting. Another weakness are relatively liberal inclusion criteria, to some extent related to the problem described above. This is partly related to the organizational heterogeneity of the study over several years. Nevertheless, the numeric criteria were combined with a need for a referral by a specialist pediatric hematologist. The relative costs of genetic diagnostics are dropping, and in our experience, several patients who, in some algorithms, could have been exempted from genetic diagnostics were shown to have *ELANE*-related SCN (19).

As demonstrated in our studies, patients with the *ELANE* gene defects may develop symptoms at a later age or learn about the mutation during family tests or after developing leukemia. These prognostic perspectives also make genetic screening beneficial for CN patients. SCN may predispose to MDS or AML, as a result of causative variants in *ELANE*, *HAX1*, *G6PC3*, *WAS*, *GATA2*, *SBDS*, *CLPB*, *CSF3R*, and the leukemia-associated genes in a majority of patients (44). Early detection of genetically conditioned neutropenia and the introduction of G-CSF therapy in the first months of life may increase the ANC and improve the clinical condition of a patient. However, in some cases, patients do not respond to the G-CSF therapy. Different forms of congenital neutropenia show granulocyte developmental abnormalities at different stages, and there is a strong correlation between the stage and severity of granulocyte developmental abnormalities and the efficacy of G-CSF therapy (45). Moreover, the cumulative dose of G-CSF may influence leukemia development. In such cases, HSCT is an appropriate therapeutic approach. It can reduce the mortality of CN patients by precluding severe infections and reducing the exposure of CN patients to G−CSF. Early HSCT is also known to lower the risk of leukemia development in patients with the *ELANE* defect (46, 47). Therefore, regular genetic screening for acquired genetic changes is necessary to monitor molecular events leading to leukemogenesis, to reduce mortality among SCN patients, and to allow physicians to establish patients’ long-term management plans and familial genetic counselling.

## Data Availability

The original contributions presented in the study are publicly available. This data can be found here: https://www.ncbi.nlm.nih.gov/clinvar?term=SUB15547179.
