# Supplementary material for: The genetic landscape of congenital neutropenia in Poland: summary of the nationwide screening campaign
Source: Front Immunol. 2025 Nov 26;16:1688208. doi: 10.3389/fimmu.2025.1688208 (PMC12689915; doi:10.3389/fimmu.2025.1688208)
Supplement: Supplementary file 1 [file DataSheet1.pdf]

Supplementary Table 1. Primers used for Sanger sequencing.

STARTER NAME	*SEQUENCE*	*ANNEALING TEMPERATURE [^{0}C]*
ELANE_F1	GTCCCCCTTCTCCCCCTTTTC	**65**
***ELANE*_R1**	GGGCTCCTGGTGGTGGCTTC	
ELANE_F2	CTTGGCAGGCACTCAGCACC	**65**
***ELANE*_R2**	CCTCCCCCCACCCACCTCA	
ELANE_F3	TGAGGTGGGTGGGGGGAG	**65**
***ELANE*_R3**	CACCCAGCCCACGATGCCAC	
ELANE_F4	CGGAGAGGGGAGGGTCATCAT	**65**
***ELANE*_R4**	TAGCCACGGTGCCTGTTGC	
ELANE_F5	CTCTGCTCCCCACCCGCTCC	**65**
***ELANE*_R5**	CACCCAATCACACAGCCAAGGAG	
GFI1_F1	GTCCCGCTCTCCCTGGCTTG	**58**
GFI1_R1	GGAAACAAAAACAGCAGCAAAAGGG	
GFI1_F2	GGGGATTGGGGGAGCCTTC	**60**
GFI1_R2	TGCCTCCTTCCCCTACGAATCAGC	
GFI1_F3	GACGCTCCCCTTGTGCCTCCA	**68**
GFI1_R3	CGGCAGGCGAGGTGGTGAG	
GFI1_F4	CTCACCACCTCGCCTGCCGT	**68**
GFI1_R4	CACCAGGGCAAGGGGACGC	
GFI1_F5	GTGTTCAAGGGATTTTAGGGTCAGC	**60**
GFI1_R5	CAAATCAAGGTGGCTCTGGGGA	
GFI1_F6	ATACTTTCTCCCCTCCCCCTCCC	**60**
GFI1_R6	TGAGGTGTAGCAGCAAAGGAGAAAGTAT	
HAX1_F1	CGGAAACTGGTCTCTGAAAGGTGG	**59**
HAX1_R1	CTTGGTTACTGTGGCGGGGTGAG	
HAX1_F2	CAGACCCCTTGCTCTTGTCCCAC	**59**
HAX1_R2	GTCTTCTCACACTTCCCATCCCCC	
HAX1_F3	GGGGATGGGAAGTGTGAGAAGAC	**59**
HAX1_R3	CTCACTTCAGGACCAGATGGGATAC	
HAX1_F4	GGATACATAAAACAGCCAGGAAGGAGTG	**59**
HAX1_R4	CCATACTTTCCTTTACCTCTTTCTTTCAAC	
HAX1_F5	GTTGAAAGAAAGAGGTAAAGGAAAGTATGG	**59**
HAX1_R5	TAGAAACTGAAATAGCCTTGACAAAACC	
G6PC3_F1	CCCTGAGGAAACGCCTTTACAA	**63**
G6PC3_R1	CCTTGAGACAGTTGCTGAAAGAGACC	
G6PC3_F2	GTCCCTTTTTGACTTCACCCCTC	**63**

G6PC3_R2	TCACCCGAACCCTAACCACATT	
G6PC3_F3-4	GTTCTGCCTCCATCTTCTCACCAC	**66**
G6PC3_R3-4	CATCCTCTCAGTCCACCCCGT	
G6PC3_F5	GTCGGGGTGGGGAGGGTC	**66**
G6PC3_R5	GCAGGCGAGATAGGACAGGG	
G6PC3_F6	GACCCTCACAGGCACAAAACAGA	**63**
G6PC3_R6	TACTGGAGCAGCCTGGCAAAG	
JAGN1_F1	GCGGAGAGGAGTGACCATAGAGT	**66**
JAGN1_R1	GCGGAACAAGGGGTGGAGC	
JAGN1_F2	CCCTCTGCTCTGCCCCACA	**66**
JAGN1_R2	TCAGCAGATGGCAAAAGGAGC	
WAS_F9	GGGCAGGAGAGGGCAAGAGG	**66**
WAS_R9	CCCACTGACCAACTCCTGACTGAAG	

Supplementary Table 2. T*he panel of 54 genes related to SCN and neutropenia associated syndromes.*

ACTB	*AK2*	*AP3B1*	*CD40LG*
CEBPE	*CLPB*	*CSF2RA*	*CSF3R*
CTSC	*CXCR2*	*CXCR4*	*DNAJC21*
DNM2	*EFL1*	*EIF2AK3*	*ELANE*
FCGR3B	*G6PC3*	*GATA1*	*GATA2*
GFI1	*GINS1*	*HAX1*	*HYOU1*
IFNGR2	*IRAK4*	*JAGN1*	*LAMTOR2*
LYST	*MKL1*	*MRTFA*	*MYD88*
PGM3	*RAB27A*	*RAC2*	*RBSN*
RMRP 3	*ROBLD*	*RUNX1*	*SBDS*
SLC37A4	*SMARCD2*	*SRP54*	*SRP72*
STK4	*TAZ*	*TCIRG1*	*TCN2*
USB1	*VPS13B*	*VPS45*	*WAS*
WDR1	*WIPF1*		

Supplementary Table 3. T*he expanded panel of genes related to inborn errors of immunity and hematological disorders.*

ACP5	*ABCB1*	*ABCB7*	*ABCG5*	*ABCG8*	*ACD*
ADAMTS13	*ACTB*	*ACTN1*	*ACVRL1*	*ADA*	*ADAM17*
AK2	*ADAR*	*ADAR1*	*AICDA*	*AIRE*	*AK1*
ANO6	*ALAS2*	*ALDOA*	*AMN*	*ANK1*	*ANKRD26*
APOC2	*AP1S3*	*AP3B1*	*AP3D1*	*APOA1*	*APOA2*
ATM	*APOC3*	*APOL1*	*ARPC1B*	*ASXL1*	*ATG16L1*
BCL11B	*ATP6AP1*	*B2M*	*BACH2*	*BAFF-R*	*BCL10*
BMPR2	*BLK*	*BLM*	*BLNK*	*BLOC1S3*	*BLOC1S6*
BTK	*BPGM*	*BRAF*	*BRCA1*	*BRCA2*	*BRIP1*
C1S	*C15orf41*	*C1QA*	*C1QB*	*C1QC*	*C1R*
C4B	*C2*	*C2BP1*	*C3*	*C3AR1*	*C4A*
C8G	*C5*	*C6*	*C7*	*C8A*	*C8B*

CARMIL2	*C9*	*CALR*	*CARD11*	*CARD14*	*CARD9*
CD247	*CASP10*	*CASP8*	*CCBE1*	*CD19*	*CD226*
CD40	*CD27*	*CD36*	*CD3D*	*CD3E*	*CD3G*
CD79A	*CD40LG*	*CD46*	*CD55*	*CD59*	*CD70*
CDCA7	*CD79B*	*CD81*	*CD8A*	*CDAN1*	*CDC42*
CFH	*CEBPA*	*CEBPE*	*CECR1*	*CFB*	*CFD*
CFI	*CFHR1*	*CFHR2*	*CFHR3*	*CFHR4*	*CFHR5*
CLCN7	*CFP*	*CFTR*	*CHD7*	*CHST14*	*CIITA*
CORO1A	*CLPB*	*COL17A1*	*COL1A1*	*COL7A1*	*COPA*
CSF2RB	*COX4I2*	*CR2*	*CRFB4*	*CSF1R*	*CSF2RA*
CTPS1	*CSF3R*	*CST3*	*CSTA*	*CTC1*	*CTLA4*
CYCS	*CTSC*	*CXCR2*	*CXCR4*	*CYBA*	*CYBB*
DIAPH1	*DBA2*	*DCLRE1B*	*DCLRE1C*	*DGKE*	*DHFR*
DOCK2	*DKC1*	*DNAJC21*	*DNASE1L3*	*DNASE2*	*DNMT3B*
EPB41	*DOCK8*	*DTNBP1*	*ELANE*	*ENG*	*ENO1*
ERCC2	*EPB42*	*EPCAM*	*EPG5*	*EPO*	*ERAP2*
F10	*ERCC3*	*ERCC4*	*ERCC6L2*	*ETV6*	*EXTL3*
F2R	*F11*	*F12*	*F13A1*	*F13B*	*F2*
FAAP24	*F3*	*F5*	*F7*	*F8*	*F9*
FANCE	*FADD*	*FANCA*	*FANCB*	*FANCC*	*FANCD2*
FAS	*FANCF*	*FANCG*	*FANCI*	*FANCL*	*FANCM*
FERMT1	*FASLG*	*FAT4*	*FCGR2C*	*FCGR3A*	*FCN3*
FLI1	*FERMT3*	*FGA*	*FGB*	*FGG*	*FLG*
FPR1	*FLNA*	*FOXC1*	*FOXC2*	*FOXN1*	*FOXP3*
GATA1	*FUT2*	*FYB*	*G6PC3*	*G6PD*	*G6PT1*
GDF2	*GATA2*	*GATA3*	*GBA*	*GCLC*	*GCS1*
GJB3	*GFI1*	*GFI1B*	*GGCX*	*GINS1*	*GJA1*
GP1BB	*GJB4*	*GLA*	*GLRX5*	*GNE*	*GP1BA*
GSS	*GP6*	*GP9*	*GPI*	*GPX1*	*GSR*
HBB	*GUCY2C*	*HABP2*	*HAX1*	*HBA1*	*HBA2*
HMOX1	*HBD*	*HBG1*	*HBG2*	*HELLS*	*HK1*
HPS4	*HOIL1*	*HOIP1*	*HOXA11*	*HPS1*	*HPS3*
HYOU1	*HPS5*	*HPS6*	*HRAS*	*HRG*	*HTRA2*
IFNGR2	*ICOS*	*ICOSLG*	*IFIH1*	*IFNAR2*	*IFNGR1*
IKBKG	*IGHM*	*IGKC*	*IGLL1*	*IKBA (NFKBIA)*	*IKBKB*
IL12RB1	*IKZF1*	*IL10*	*IL10RA*	*IL10RB*	*IL12B*
IL18	*IL12RB2*	*IL17A*	*IL17F*	*IL17RA*	*IL17RC*
IL2RA	*IL18RAP*	*IL1RN*	*IL21*	*IL21R*	*IL23R*
IL7	*IL2RG*	*IL36RN*	*IL5*	*IL6*	*IL6R*
IRF1	*IL7R*	*INAVA*	*INO80*	*IRAK1*	*IRAK4*
IRF8	*IRF2BP2*	*IRF3*	*IRF4*	*IRF5*	*IRF7*
ITGA3	*IRGM*	*ISG15*	*ITCH*	*ITGA2*	*ITGA2B*
JAGN1	*ITGAM*	*ITGB2*	*ITGB3*	*ITK*	*ITM2B*
KDSR	*JAK1*	*JAK3*	*KCNK3*	*KCNN4*	*KDM6A*
KNG1	*KIF23*	*KLF1*	*KLKB1*	*KMT2A*	*KMT2D*
LCK	*KRAS*	*KRT1*	*KRT10*	*LAMTOR2*	*LAT*
LRRC8A	*LIG1*	*LIG4*	*LMAN1*	*LPIN2*	*LRBA*
MALT1	*LYN*	*LYST*	*LYZ*	*MAD2L2*	*MAGT1*
MCP	*MAP3K14*	*MASP2*	*MASTL*	*MCFD2*	*MCM4*
MPL	*MECOM*	*MEFV*	*MKL1*	*MOGS*	*MPIG6B*
MSN	*MPO*	*MRE11*	*MRE11*	*MS4A1*	*MSH6*
MYH9	*MTHFD1*	*MTR*	*MTRR*	*MVK*	*MYD88*
NCF1	*MYO5B*	*MYSM1*	*NBAS*	*NBEAL2*	*NBN*
NFKB1	*NCF2*	*NCF4*	*NCSTN*	*NEMO*	*NFAT5*
NLRP1	*NFKB2*	*NFKBIA*	*NHEJ1*	*NHP2*	*NLRC4*
NRAS	*NLRP12*	*NLRP3*	*NOD2*	*NOP10*	*NOTCH3*
OTULIN	*NSMCE3*	*NT5C3A*	*OFD1*	*ORAI1*	*OSTM1*
PGK1	*P2RY12*	*PALB2*	*PARN*	*PARN*	*PEPD*
PIK3CD	*PGM3*	*PHF9*	*PIEZO1*	*PIGA*	*PIGA*
PLAU	*PIK3R1*	*PKLR*	*PKLR*	*PLA2G4A*	*PLAT*
PNP	*PLCG2*	*PLEC*	*PLEKHM1*	*PLG*	*PMS2*
PRKACG	*POLA1*	*POLE*	*POLE2*	*POMP*	*PRF1*
PSENEN	*PRKCD*	*PRKDC*	*PROC*	*PROS1*	*PSEN*
PTEN	*PSMA3*	*PSMB4*	*PSMB8*	*PSMB9*	*PSTPIP1*
RAC2	*PTPN11*	*PTPN22*	*PTPRC*	*PUS1*	*RAB27A*
RANBP2	*RAD50*	*RAD51A*	*RAD51C*	*RAG1*	*RAG2*
RBM8A	*RASGRP1*	*RASGRP2*	*RB1*	*RBCK1*	*RBCK1*
RFXANK	*RECQL4*	*RELB*	*RET*	*RFWD3*	*RFX5*
RMRP	*RFXAP*	*RHAG*	*RHOH*	*RIPK1*	*RLTPR*
RPL26	*RNASEH2A*	*RNASEH2B*	*RNASEH2C*	*RNF168*	*RNF31*
RPS17	*RORC*	*RPGR*	*RPL11*	*RPL15*	*RPL18*
RPS29	*RPL27*	*RPL35*	*RPL35A*	*RPL5*	*RPS10*

SAMD9	*RPS19*	*RPS24*	*RPS26*	*RPS27*	*RPS28*
SEC23B	*RPS7*	*RPSA*	*RTEL1*	*RUNX1*	*RUNX3*
SERPINF2	*SAMD9L*	*SAMHD1*	*SAMHD1*	*SBDS*	*SCN1A*
SLC11A2	*SEMA3E*	*SERPINA10*	*SERPINC1*	*SERPIND1*	*SERPINE1*
SLC39A4	*SERPING1*	*SGPL1*	*SH2D1A*	*SH3BP2*	*SKIV2L*
SLX4	*SLC19A2*	*SLC25A38*	*SLC29A3*	*SLC35C1*	*SLC37A4*
SNX10	*SLC45A2*	*SLC46A1*	*SLC4A1*	*SLC7A7*	*SLFN14*
SRC	*SMAD1*	*SMAD4*	*SMAD9*	*SMARCAL1*	*SMARCD2*
STAT4	*SOS1*	*SP110*	*SPINK5*	*SPTA1*	*SPTB*
STN1	*SRP54*	*SRP72*	*STAT1*	*STAT2*	*STAT3*
TAP2	*STAT5B*	*STAT6*	*STEAP3*	*STIM1*	*STK4*
TBXA2R	*STX11*	*STXBP2*	*SUOX*	*TACI*	*TAP1*
TERT	*TAPBP*	*TAZ*	*TBK1*	*TBX1*	*TBX21*
TGFB3	*TBXAS1*	*TCF3*	*TCIRG1*	*TCN2*	*TERC*
TIRAP	*TF*	*TFPI*	*TFRC*	*TGfb1*	*TGFB2*
TMEM173	*TGFBRII*	*THBD*	*THPO*	*TICAM1*	*TINF2*
TNFRSF13C	*TLR2*	*TLR3*	*TLR5*	*TMC6*	*TMC8*
TNFSF12	*TMEM50B*	*TMPRSS6*	*TNFAIP3*	*TNFRSF11A*	*TNFRSF13B*
TPM4	*TNFRSF1A*	*TNFRSF4*	*TNFRSF5*	*TNFRSF6*	*TNFSF11*
TRAF6	*TNFSF15*	*TNFSF5*	*TNFSF7*	*TP53*	*TPI1*
TUBB1	*TPP1*	*TPP2*	*TRAC*	*TRAF3*	*TRAF3IP2*
UNG	*TREX1*	*TRNT1*	*TSR2*	*TTC37*	*TTC7A*
VPS13B	*TYK2*	*UBE2T*	*UNC119*	*UNC13D*	*UNC93B1*
WRAP53	*UROS*	*USB1*	*USP18*	*VIPAS39*	*VKORC1*
ZBTB24	*VPS45*	*VWF*	*WAS*	*WDR1*	*WIPF1*
ZMIZ1	*XIAP*	*XRCC2*	*XRCC9*	*YARS2*	*ZAP70*

Supplementary Table 4. Clinical data and screening results for patients with genetically confirmed diagnosis including the scores for variants of unknown significance.

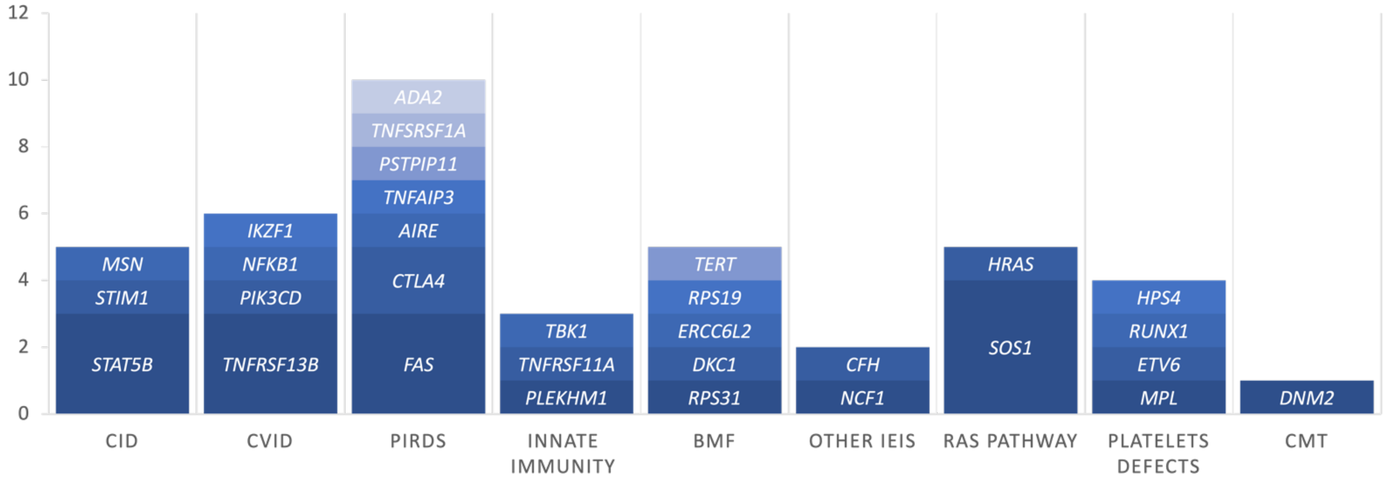

Supplementary Figure 1. *Distribution of disorders and genetic variations revealed by reanalysis in panel of genes associated with IEI and hematological diseases.*
